# Filling the Gaps: Using Synthetic Low-Altitude Aerial Images to Increase Operational Design Domain Coverage

**DOI:** 10.3390/s24041144

**Published:** 2024-02-09

**Authors:** Joachim Rüter, Theresa Maienschein, Sebastian Schirmer, Simon Schopferer, Christoph Torens

**Affiliations:** German Aerospace Center (DLR), Institute of Flight Systems, 38108 Braunschweig, Germany; theresa.maienschein@dlr.de (T.M.); christoph.torens@dlr.de (C.T.)

**Keywords:** operational design domain (ODD), environment perception, object detection, machine learning, synthetic images, game engine, unmanned aerial vehicle (UAV), unmanned aerial system (UAS)

## Abstract

A key necessity for the safe and autonomous flight of Unmanned Aircraft Systems (UAS) is their reliable perception of the environment, for example, to assess the safety of a landing site. For visual perception, Machine Learning (ML) provides state-of-the-art results in terms of performance, but the path to aviation certification has yet to be determined as current regulation and standard documents are not applicable to ML-based components due to their data-defined properties. However, the European Union Aviation Safety Agency (EASA) published the first usable guidance documents that take ML-specific challenges, such as data management and learning assurance, into account. In this paper, an important concept in this context is addressed, namely the Operational Design Domain (ODD) that defines the limitations under which a given ML-based system is designed to operate and function correctly. We investigated whether synthetic data can be used to complement a real-world training dataset which does not cover the whole ODD of an ML-based system component for visual object detection. The use-case in focus is the detection of humans on the ground to assess the safety of landing sites. Synthetic data are generated using the methods proposed in the EASA documents, namely augmentations, stitching and simulation environments. These data are used to augment a real-world dataset to increase ODD coverage during the training of Faster R-CNN object detection models. Our results give insights into the generation techniques and usefulness of synthetic data in the context of increasing ODD coverage. They indicate that the different types of synthetic images vary in their suitability but that augmentations seem to be particularly promising when there is not enough real-world data to cover the whole ODD. By doing so, our results contribute towards the adoption of ML technology in aviation and the reduction of data requirements for ML perception systems.

## 1. Introduction

For Unmanned Aircraft Systems (UAS) to reach high levels of autonomy, they need exceptional environment perception capabilities. On the one hand, to perform their nominal mission independently and safely, e.g., to detect obstacles or other aircraft to avoid, to detect dropping zones for cargo delivery or to detect objects to be observed or monitored. On the other hand, environment perception is crucial for the autonomous execution of contingency and emergency procedures, e.g., to assess unprepared emergency landing sites after a critical system failure. Artificial Intelligence (AI) and more precise Machine Learning (ML) currently produce state-of-the-art results in many semantic environment perception tasks, and therefore, are a key enabling technology for UAS to reach these high levels of autonomy.

However, in aviation, high safety and assurance requirements pose a major challenge for the integration of ML-based systems. The development standards and assurance techniques that have been established for decades simply do not allow the behavior of a system to be defined by data and by models whose internal workings are opaque to humans.

The European Aviation Safety Agency (EASA) has recognized this problem and is currently working on concepts, guidelines and standards that shall help to integrate ML into avionic systems. In one of their recent publications, the EASA introduces a trustworthiness analysis that shall be carried out first during the development process of an ML system [1]. The first step during this analysis is the identification of the operational environment in which the ML-based system will be operated. Next, the identified parameters of this operational environment shall be formally captured within an Operational Design Domain (ODD). During training, the ODD shall be used for learning assurance by assessing whether the data used for training are *complete* with respect to the ODD. Here, completeness means that the data “sufficiently […] covers the entire space of the operational design domain of the intended application” [1]. Unfortunately, in aviation, it is often difficult to collect a large amount of training data with a high degree of completeness, especially for environment perception tasks. This is because recording a large amount of real flight data is very expensive and, in certain cases, too dangerous. For example, we must consider close encounters with other aircraft or flying nearby high voltage power-lines or forest fires.

EASA also mentions ways to circumvent this problem, one of them being the use of synthetic training data. It is known that training on synthetically generated data can improve the general performance of visual ML models slightly in some use-cases [2,3,4]. Improvements have also been shown for rare classes where little data are available [5]. However, usually there is only a very slight improvement in model performance and, to the best of our knowledge, if and how synthetic data can help to improve the coverage of an ODD when not enough real-world data are available has not been studied. Since there are usually not enough real-world data to cover the whole ODD and because of the problems explained above, it is an important but open question whether the different types of synthetic data presented by EASA are really suitable for increasing the ODD coverage of an ML model. The answer to that question will have a huge impact on the difficulty of deploying ML models in aviation.

In this work, we build upon these concepts and examine if and how synthetic data can be used to increase the completeness of a dataset with respect to the ODD. Our examination is based on an ML-based detection system that shall detect humans on the ground. For example, such a system can be used to assess the clearance of a potential contingency landing site. For our evaluation, we focus on one of the central ODD parameters for such a detection task, namely the flight altitude. In correspondence with the available dataset, it is assumed that the UAS is operated over a grassy area at an altitude of 4–103 m above the ground. Further, it is assumed that real-world images are only available for a range of altitude from 4 to 70 m. Therefore, synthetic images that represent an altitude of 70–103 m are used to increase the coverage of this ODD dimension. This altitude is chosen for the purpose of this research and may change for other practical applications in accordance with given boundary conditions. We generate data for different kinds of synthetic data mentioned by EASA, mix them into the training set and evaluate their influence on the performance of a state-of-the-art model for object detection using a real-world test dataset. We present the evaluation results and discuss the benefits, challenges and open problems of using synthetic training data for increasing the coverage of the ODD in this UAS use-case. To the best of our knowledge, this is the first paper to compare and evaluate the EASA classes of synthetic data in the context of ODD.

Our main contributions are as follows. On the one hand, we give a condensed overview of the new EASA documents regarding the introduction of ML in aviation, with a special focus on ODD and synthetic data. We relate both topics to their use in aviation. On the other hand, we present approaches to generating different types of synthetic data according to the EASA, name the problems and evaluate the usefulness of them for the use-case of ODD coverage for human detection.

## 2. Related Work

Data-driven approaches like Machine Learning rely on large training datasets that are both representative and complete—properties that are often hard to achieve. Some papers even find that “the performance on vision tasks increases logarithmically based on volume of training data size” [6]. One active field of research that promises to reduce the amount of real-world data needed is the use of synthetic data. Synthetic data can be generated in different ways that range from basic geometric transformations of real images to the extraction of rendered images from game engines. An in-depth introduction into synthetic data and their applications can be found, e.g., in [7]. While basic transformations like changing contrast and brightness, applying geometric transformation, etc., that are described, e.g., in [8], are used in most ML training procedures, the following will focus on more advanced types of synthetic images.

In the automotive industry, synthetic data have been used to detect other vehicles or pedestrians using visual simulation environments [3,9,10,11] where the camera perspective is close to the ground. In contrast to this, in aviation, the camera perspective is birds-eye. However, the tools used to generate synthetic data are similar and synthetic data have been used to train Machine Learning models for human detection [12], car detection [13], animal detection [14] and semantic segmentation [2].

Results generally show that synthetic data alone is not enough to train an ML model that is able to generalize to real-world data because of the so-called *domain shift*, see, e.g., [4,9,11,15]. However, when used in combination with real-world data, synthetic data may be able to improve the overall model performance [3,9,11,14,15,16] as well as its performance on rare classes [5]. Furthermore, there is a large body of work on reducing performance drops from all forms of domain shift called *domain adaption* where promising results have been achieved. For a survey paper see [17].

Overall, the impact of the domain shift varies and remains hard to predict for different domains and use-cases. In this paper, we investigated whether synthetic data can be used to fill gaps in the intended operational design domain that state the limits of the operation.

## 3. AI in Aviation

In this section, the current efforts to introduce ML to avionic systems are presented. This is followed by a focus on the different classes of synthetic data that can be used to train an ML system when there are not enough real-world data available.

### 3.1. AI Assurance in Aviation

In 2020, the *European Union Aviation Safety Agency* (EASA) published the *AI Roadmap* [18], which presents a foundation for the future usage of Artificial Intelligence (AI) in aviation, in particular, Machine Learning (ML). They observe that “traditional development assurance frameworks are not adapted to machine learning” [18] and identify four “Trustworthy AI Building Blocks” which should increase trust in ML components and ultimately enable their use in aviation. These blocks are called *Trustworthiness Analysis*, *Learning Assurance*, *Explainabilty* and *Safety Risk Mitigation*. The Trustworthiness Analysis block primarily focuses on ethical issues and encompasses the seven gears of the European Union ethical guidelines, namely “accountability, technical robustness and safety, oversight, privacy and data governance, non-discrimination and fairness, transparency, societal and environmental well-being”. The Learning Assurance block is based on the observation that existing development processes are not suitable for ML. Therefore, it concludes that a new development assurance process has to be implemented which shifts its focus to data correctness and completeness, novel verification methods, etc. The process is introduced and explained in subsequently published documents [19,20]. Explainability is a “human-centric” concept and deals with the problem that artificial neural networks are black-box models and humans cannot comprehend the internal processes. This block is intended to soften this and aims to provide explanations that are understandable to humans. In the Safety Risk Mitigation block, it is assumed that the ML black box cannot always be opened to a sufficient extent, and that, therefore, monitoring and risk mitigations are needed. This can be carried out, for example, with conventional methods, such as object tracking in the object recognition use-case, or by monitoring certain parameters and thresholds [20]. These four blocks are intended to form the basis of the analysis and certification of AI systems. They are refined in the subsequently published documents ”Concepts of Design Assurance for Neural Networks” (CoDANN) 1 and 2 [19,20] and the theoretical backgrounds of the problems are explained. A more in-depth review of these document can be found in, e.g., [21].

Furthermore, in 2023, the EASA published a document [22] that first defines concrete objectives that should be fulfilled to develop trustworthy AI. In this context, the EASA adopts the terms *Operational Domain* (OD) and *Operational Design Domain* (ODD) from the automotive domain. For the automotive domain, the formal definition of ODD is given by *SAE J3016* [23]. For the aviation domain, the concept of OD is newly introduced: “Operating conditions under which a given AI-based system is specifically designed to function as intended, in line with the defined ConOps, including but not limited to environmental, geographical, and/or time-of-day restrictions” [22]. The *concept of operations* (ConOps) is of central importance within aviation, specifically as part of the EASA *specific* category [24,25]. It serves as an informal document and a basis for operational risk assessments. In addition to this, the EASA defines the ODD as follows: “The ODD defines the set of operating parameters, together with the range and distribution within which the AI/ML constituent is designed to operate, and as such, will only operate nominally when the parameters described within the ODD are satisfied. The ODD also considers dependencies between operating parameters in order to refine the ranges between these parameters when appropriate; in other words, the range(s) for one or several operating parameters could depend on the value or range of another parameter” [22]. Note that the EASA differentiates between the system level (OD) and the AI/ML constituent level (ODD).

Hence, an ODD captures the nominal input space that the developed ML system will be faced with during operation and effectively limits the foreseeable input space of a given ML-based system within its designated use-case scenarios. By defining the ODD, the developer and the certification authorities have created a defined input space in which the model has to be tested and has to work as expected. Furthermore, when the input to the ML component is monitored during operation, the system can decide whether the ML component is designed to operate on the input data or whether its output cannot be trusted and therefore should not be used. This ultimately increases the safety of the overall system.

### 3.2. Synthetic Training Data

The development of an ML application often requires a lot of data for training, validation and testing. For some applications, however, there are not enough real-world data available. This is especially true in aviation, where the operation of UAS to generate large amounts of data is expensive and, for some situations, dangerous or even impossible. Synthetic data promise to compensate this lack of data. In case of missing data to cover the ODD, the EASA proposes to use data augmentation or even synthetic data to fill the gaps [22]. In the report *Concepts of Design Assurance for Neural Networks* (CoDANN) [19], the EASA gives more details about the work with synthetic data. First, synthetic data are defined as “any data that was computer-generated or any data from the target sensors that underwent a processing step that is not included in the target operational system” [19]. Then, synthetic data are classified into three different classes, distinguished by the way of creation.

Class-1 contains synthetic data created by “basic transformations of real data” [19]. This includes geometric transformations like translations, rotations, scaling, as well as transformations of image attributes like brightness and noise. These transformations are commonly known as data augmentations.

Class-2 contains synthetic data created using “more advanced transformations of real data” [19]. This class is characterised by not just transforming the existing pixels but e.g., recomposing multiple images. For example, pictures of real objects could be pasted on other real background pictures.

Class-3 contains “fully or mostly synthetic data” generated without the use of real data [19]. Data based on 3D graphics rendering fall into this class. To create Class-3 synthetic data, computer game engines or other rendering tools can be used.

## 4. Data

In this section, the different kinds of synthetic data used in the experiments as well as the processes to generate the data are described.

### 4.1. Real Training and Evaluation Data

As a real-world dataset, the publicly available PeopleOnGrass dataset [26] is used. The dataset shows people on mostly grassy areas from various angles and altitudes between 4 and 103 m. It contains 13,713 objects in 2900 images. The images are taken with a resolution of 3840 × 2150 px and also come with meta data like GPS location as well as UAS altitude and attitude.

**Figure 1:**
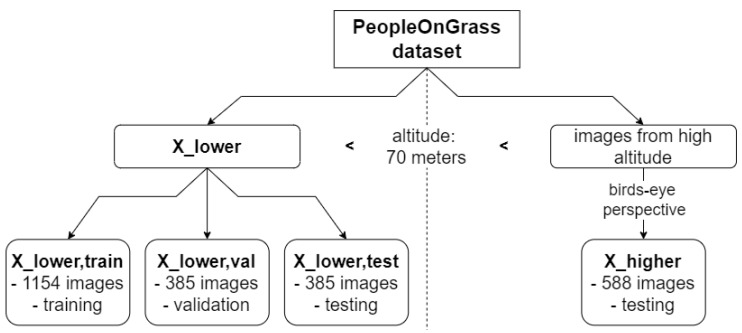
Splitting of the PeopleOnGrass dataset into training, validation and testing datasets according to the experimental ODD design.

**Figure 2:**
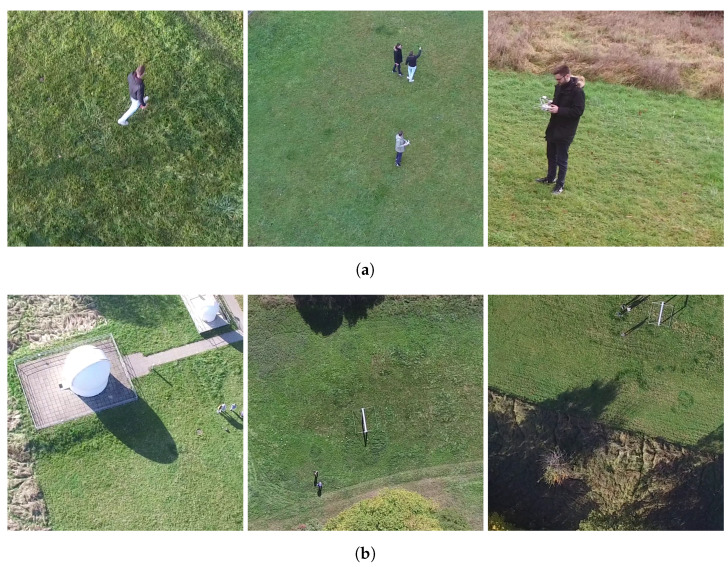
Examples of the real-world images from the PeopleOnGrass dataset [26]. Images are cropped to the dimensions needed for the used Machine Learning model. (**a**) Real images from the PeopleOnGrass dataset [26] taken at an altitude of lower than 70 m. (**b**) Real images from the PeopleOnGrass dataset [26] taken at an altitude of higher than 70 m and with a birds-eye perspective.

In this work, altitude is considered as the ODD parameter for which the coverage should be increased. Usually, an ODD has many parameters whose consideration would increase the complexity drastically. This simplification is made to be able to investigate the influence of the addition of the synthetic data in isolation. To conduct the experiments, this dataset is split and images taken at an altitude lower than 70 m are considered as suitable for training. The images taken at an altitude of more than 70 m and from birds-eye perspective (70°–90° [26]) are considered as the ODD space which should be covered with synthetic images. The corresponding real images will only be used for testing the models. The dataset used for training will be called $X_{\mathrm{lower}}$. It contains 1924 images. The dataset containing images at an altitude of more than 70 m and from birds-eye perspective will only be used for testing and will be called $X_{\mathrm{higher}}$. It contains 588 images. Furthermore, $X_{\mathrm{lower}}$ is split into a training, a validation and a test dataset, called $X_{\mathrm{lower},\mathrm{train}}$, $X_{\mathrm{lower},\mathrm{val}}$ and $X_{\mathrm{lower},\mathrm{test}}$ which contain 1154, 385 and 385 images, respectively. The complete splitting process is visualized in Figure 1. Example images are shown in Figure 2.

### 4.2. Class-1 Synthetic Training Data

Since we use different augmentation techniques during training by default, only scaling, cropping and the mixing together of image patches, similar to CutMix [27] and Mosaic [28], are used to create Class-1 data. The overall goal is to downscale and combine the images taken at an altitude of less then 70 m to make them look as if they were recorded at an altitude of more than 70 m.

**Figure 3:**
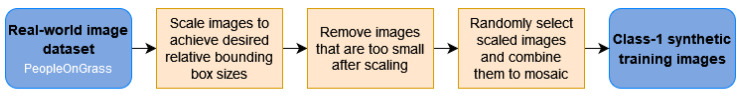
Process for generating Class-1 synthetic training images.

In order to scale the images with acquisition heights of less than 70 m, the first step is to calculate suitable scaling factors. To do so, the relative bounding box areas in the real-world images for heights between 70 and 103 m are evaluated. The upper and lower quartiles are used as references for the scaling of the Class-1 training data. For each image taken at altitudes below 70 m, a scaling factor is calculated for which the bounding box areas of all objects would be within the desired range after scaling. If any dimension of the scaled image is smaller than 350 px, the image is discarded as it becomes too pixelated. Since the images need to have at least 800 × 800 px for the training and only 346 scaled images fulfill this requirement, different images are combined in the last step. To do so, a script randomly chooses images and merges them together to create a single 800 × 800 px image. Different to CutMix [27] or Mosaic [28] images, we do not use a specific ratio to cut the images. Instead, the images are cut at random positions to ensure an even bigger variance in visual appearance. Furthermore, we do not fix the number of images that are put together. Instead, one to four images are put together into one image depending on their size. As the bounding boxes of the original images are known, the labels of the final Class-1 images can be calculated automatically. Figure 3 shows the general procedure used to create the Class-1 training data. In total, 2800 Class-1 training images are created.

The advantage of Class-1 synthetic images is that they resemble the real-world images very closely, as the synthetic images are generated by transforming real-world images using augmentations. That makes the synthetic images look very realistic. However, the need for the real-world images is also a strong limitation of this approach. As only the existing real-world images are transformed, the variation that can be introduced is limited. Furthermore, this approach can only be applied if real-world data are available that can be transformed so that it represents the missing data.

### 4.3. Class-2 Synthetic Training Data

To generate Class-2 synthetic images, human images from a birds-eye perspective, called foreground images, are patched onto real-world background images from a birds-eye perspective. For the creation, two real-world aerial image datasets are used. On the one hand, a dataset that was acquired from the DLR research project *Drones4Good* is used. This dataset contains 314 images with a total number of 33,839 people. On the other hand, the Heridal dataset [29] is used. This contains over 1500 labeled aerial photographs of people in different environments like forests, parks, snowy landscapes, etc.

For the creation of the foreground images, only the internal DLR dataset is used. Using *GIMP* [30], 52 of the people pictured in various positions like standing, sitting and walking are cut out. To ensure an even bigger variety, these patches are augmented by rotating them 15°, 30°, 45°, 60°, 75° and 90° clockwise. Additionally, *imgaug* [31] is used for further augmentations like flipping the images horizontally and vertically, as well as applying GammaContrast. For the background images, both datasets are used. Two main criteria are applied to decide if an image is suitable. Firstly, the image should not show any humans, and secondly, the images should show different landscapes like forests, lakes, sidewalks, etc. Similar to the foreground images, the background images are augmented using imgaug by applying flips, GammaContrast and SigmoidContrast.

**Figure 4:**
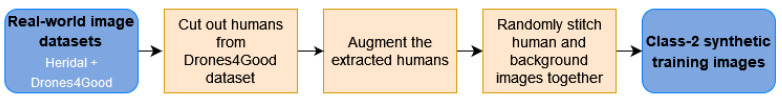
Process for generating Class-2 synthetic training images.

In the last step, a script is implemented to randomly stitch these images together. The resulting training images show between 0 and 15 people per image and the corresponding labels are generated automatically as the stitching positions are known by the script. The size of the objects in the images are fitted to the size of the bounding boxes in the real-world data as described for the Class-1 images above. The whole process is visually represented in Figure 4. Again, 2800 Class-2 images are created to represent images taken from heights between 70 and 103 m.

An advantage of Class-2 synthetic images is that they allow us to introduce more variation than Class-1 synthetic images. For example, using the stitching approach, it is possible to create images with humans in different geographical locations or in different poses. However, this approach still needs real-world images that contain this variation. In the given examples, background images of the desired geographical region are needed or foreground patches of humans in the specified poses. A further disadvantage is that the generated images may show artefacts like unrealistic sharp edges from the stitched elements. Furthermore, the stitched humans do not have shadows. Without specifying regions where the objects are allowed to be placed for each image, it is further possible that the objects are stitched to unrealistic locations, e.g., a human standing on water. It remains unclear, however, how important these factors are and how large their influence is, if any.

### 4.4. Class-3 Synthetic Training Data

To generate Class-3 synthetic images, we build on the previous work by [12] which includes a tool chain to generate synthetic images for human detection from the UAS perspective using a game engine. It is based on the *Unreal Engine* [32] 4, which is used to create and simulate virtual environments in which virtual humans can be placed. The synthetic world and synthetic humans are downloaded from the *Unreal Engine Marketplace*. To extract images, the tool *AirSim* [33] is used. AirSim is a plugin for the Unreal Engine 4 to simulate drone flights and car driving. It allows you to mount different virtual sensors like a camera and lidar to a drone or a car. The virtual sensors’ data can be accessed via a dedicated API. AirSim also enables the user to extract segmentation images which classify the shown pixels to specific object classes. These are used to generate the bounding boxes for the objects of interest.

**Figure 5:**
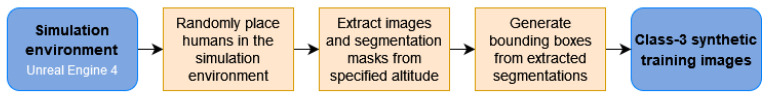
Process for generating Class-3 synthetic training images.

**Figure 6:**
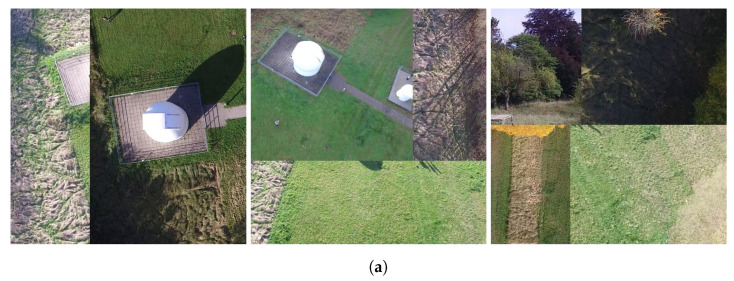

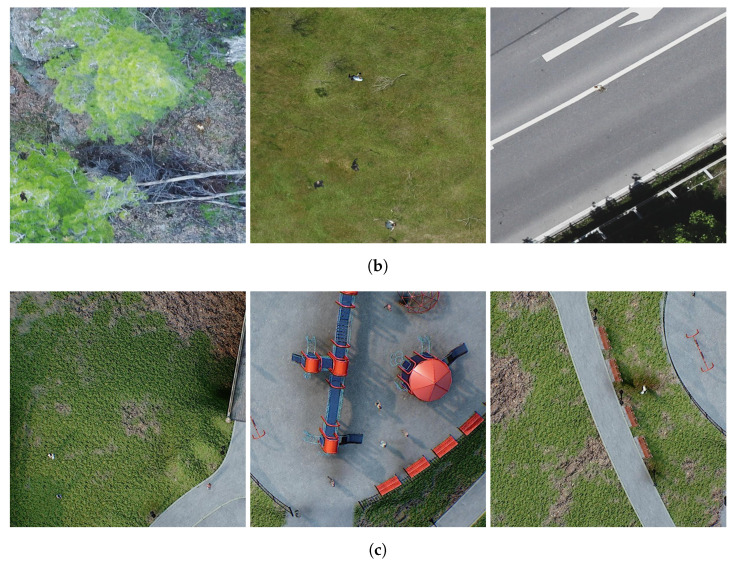
Examples of the generated synthetic images for each class. (**a**) Class-1 synthetic images generated by scaling, cropping and mixing image patches. (**b**) Class-2 synthetic images generated by stitching human patches into background images. (**c**) Class-3 synthetic images generated using a game-engine.

As [12] mentions problems with the label generation of objects that are occluded, which ultimately lead to bad object detection results, we remove most trees and water areas which could lead to humans getting occluded and thus being only partially or not visible in the image. We also disable some gravity settings within the Unreal Engine, as the virtual humans often fall and lay on the ground when they are placed on an inclined plane which would lead to a significant class imbalance. After placing the humans automatically in the environment using the tool chain, it is used to extract images with the same resolution as the real images and the field-of-view as taken from the datasheet of the camera. The images are taken from an altitude of 70–103 m and a camera angle of 70°–90° degrees, i.e., birds-eye perspective. Figure 5 shows the simplified process to generate the Class-3 synthetic images and Figure 6c shows examples of the extracted images.

The main advantage of Class-3 synthetic images is that they allow you to introduce a huge amount of variation without needing real-world images. In theory, by using a simulation environment, images of almost all situations can be generated. However, as the situation is only generated in a simulation environment, the images look less realistic than Class-1 and Class-2 synthetic images and may be discriminated from real-world images quite easily. This may be a problem for the ML model as described in Section 3.2. Furthermore, as mentioned in [12], there may also be problems with the correct placement of objects and resulting occlusions when the generation of the situation is automated.

## 5. Experiments and Results

To evaluate whether synthetic images with altitude ranges not covered by the real-world data improve the model performance, a Faster-RCNN with a ResNet-50-FPN backbone pre-trained on COCO [34] is trained. It is based on [35] and its publicly available *torchvision* implementation [36] is used. As the available real-world images are relatively large and the humans are proportionally very small, the images are cropped to 800 × 800 px. During training, random crops are extracted from the images. During evaluation, center crops are generated to always evaluate on the same image crops.

To make the trained model more robust and to mitigate some potential differences in low-level image metrics between the different kinds of synthetic and the real-world images, the standard augmentation strategies brightness and contrast change, Gauss and ISO noise, as well as blur, provided by the library *Albumentations* [37], are used. Furthermore, random horizontal flipping is applied to increase the size of the training set.

Using these augmentations, the model is trained with a batch-size of four images for a maximum of 100 epochs to make sure that the model is able to converge to a good solution. To reduce the computational load, early stopping with a patience of five is used to stop the training when the model converged before finishing 100 epochs. As optimizer, Adam [38], with a learning rate of 0.00001, is used.

To evaluate the models, the widely used metric *Average Precision* (AP) at an *Intersection over Union* (IoU) threshold of 0.5 (AP@[IoU=0.5]) from the COCO evaluation [34] is used. It measures the accuracy of the detections of the model by comparing the overlap between predicted bounding boxes and ground truth boxes. In this metric, the predicted bounding box is considered accurate if it overlaps with the ground truth box by at least 50%. This metric provides a single score to evaluate the overall performance of an object detection model by considering precision-recall curves, where precision is the ratio of true positive detections to the total number of detections, and recall is the ratio of true positive detections to the total number of ground truth objects. To compensate for the randomness in the training process, the model is trained three different times with the given parameters and the average of the AP of each trained model is taken. When not explicitly stated otherwise, the given results are always averaged over three training runs.

As we only evaluate the ML component to investigate the influence of the ODD coverage, we consider the described ML evaluation metrics. We note, however, that when looking at the performance of the whole aircraft system, these metrics would have to be incorporated into more high-level metrics at the aircraft system level, which can provide evidence for the system’s performance given the specific parameters of the aircraft.

**Figure 7:**
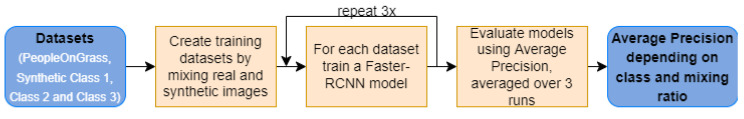
Simplified flowchart of the training and evaluation process used in this work.

To be able to evaluate whether the amount of the synthetic data added to increase the ODD coverage has an influence on the model performance, the experiments are also run with different amounts of synthetic images. The sizes are indicated using the field “Fraction of added synthetic data” in the evaluation Table 1 and Table 2. For example, as $X_{\mathrm{lower},\mathrm{train}}$ contains 1154 images, a fraction of added synthetic data of 0.1 means that 115 synthetic images are mixed into the real training dataset to increase ODD coverage. The same fraction is added to the validation set used for early stopping. For comparison, a model trained without added synthetic images is shown as a baseline. All models are trained with the parameters given above. Figure 7 shows a simplified flowchart of the training and evaluation process.

**Figure 8:**
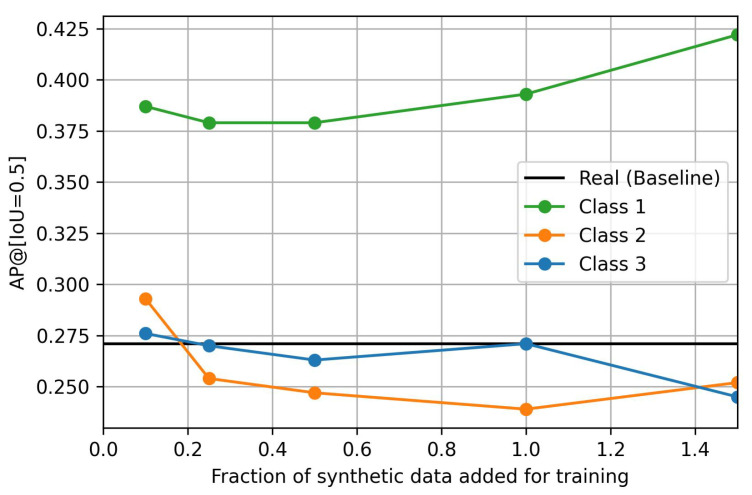
AP@[IoU=0.5] of the models on the real-world images captured at an altitude higher than 70 m. Results are averaged over 3 training runs. Dots represent results; the lines are interpolations.

Table 1 shows the AP@[IoU=0.5] on the test set $X_{\mathrm{higher}}$, i.e., the images from the PeopleOnGrass dataset taken at an altitude higher than 70 m and from a birds-eye perspective. A graphical representation of the results is shown in Figure 8. The results show that the addition of Class-1 synthetic data improves the results drastically compared to the benchmark model trained on images taken below 70 m. The general trend suggests that the addition of more data may increase the performance even further. On the other hand, the addition of a small amount of Class-2 and Class-3 data also bring slight improvements, however, these are much smaller and may even be neglected. The addition of larger subsets shows no influence or even worsens the results.

As the synthetic images should not deteriorate the performance of the model on images for which real data were available during training, the performance of the model is also evaluated on the test set $X_{\mathrm{lower},\mathrm{test}}$. The results of the models trained with the mixed datasets are shown in Table 2. They show that, at the altitude for which real-world training images were available, the addition of Class-1 synthetic images does not lead to worse performance but may instead even improve the results slightly compared to the baseline. Class-2 and Class-3 synthetic data only have a minor effect here with a tendency for a slightly lower performance.

While Table 1 and Table 2 already show clear indications of the usefulness of Class-1 synthetic data, we further investigated whether these results are based mainly on the *synthetic training data* or on the *real test data*. On the one hand, it seems reasonable to argue that higher classes of synthetic data should lead to better model performance as they are able to generate more variations in the data. On the other hand, for the given use-case of increasing the dataset’s completeness regarding the altitude, the images in $X_{\mathrm{lower}}$ and $X_{\mathrm{higher}}$ look quite similar, as shown in Figure 2, and only differ in aspects like the bounding box sizes. Therefore, the good results for Class-1 synthetic data must be attributed to their high similarity with the test data. It must be noted that these results may be specific to the ODD parameter considered in this work and may not translate to cases where the coverage of other ODD parameters shall be increased.

**Figure 9:**
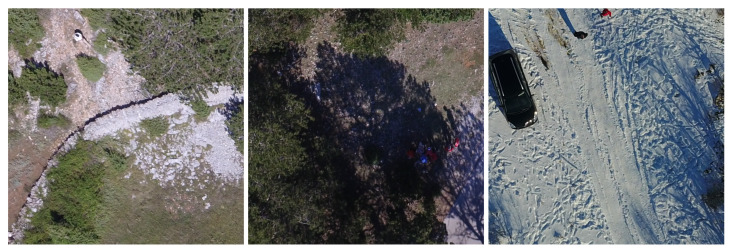
Examples of cropped images from the Heridal dataset [29] used for further evaluation.

To address this concern, the models are also trained using only the synthetic data without mixing with the real $X_{\mathrm{lower}}$ and are tested on another real-world dataset. In this case, a subset of the Heridal dataset [29] is used. In contrast to the PeopleOnGrass dataset, the Heridal dataset does not contain information about the altitude from which images were taken. Therefore, images with a similar bounding box size to the images in $X_{\mathrm{higher}}$ are selected. From these, the images are cropped to the needed size of 800 × 800 px. If there were several people in one image, several 800 × 800 px parts of the image are cut out, each containing at least one person. In total, 60 images for validation are created. Figure 9 shows some examples of the resulting images. The models are trained with the same training parameters as before and the results are again averaged over three training runs.

The model results are shown in Table 3. It can be seen that the results now clearly differ. Now, the model trained with Class-1 synthetic data only reaches half the performance of the model trained with Class-2 synthetic data, which performs the best. The model trained with Class-3 synthetic data is rated second-best and performs slightly worse than the one trained with Class-2 synthetic data. Therefore, it is reasonable to assume that the Class-1 synthetic data give the best results for the initial tasks because these are the most similar to the target domain. Should the coverage of other ODD parameter be improved, the other classes of synthetic data may lead to better results.

## 6. Discussion

In this section, we discuss various outcomes of the experiments: whether altitude is a reasonable ODD parameter, findings from the generation of the synthetic images, which synthetic data class works best in which situation, how to use synthetic data to increase the ODD coverage, how domain fitting and overfitting might affect the experiments and limitations of the approach.

### 6.1. Altitude as an ODD Parameter for UAS’ Visual Object Detection Tasks

When testing the baseline model trained on images taken at an altitude of less than 70 m on images taken under the same conditions but at a slightly higher altitude, a sharp drop in AP@[IoU=0.5] from 0.56 to 0.27 can be seen in Table 1 and Table 2. We therefore conclude that altitude is a reasonable parameter for the ODD of the considered use-case. This parameter is also relatively easy to measure and monitor which is good for practical applications.

### 6.2. Generation of Synthetic Images

The generation of Class-1 synthetic images proved to be the easiest way to generate synthetic data for the considered use-case. By using available libraries as explained above, the real-world images can be transformed to represent images taken at a different altitude.

The generation of Class-2 synthetic images required more effort as another real-world dataset to provide the background images has to be found or recorded. Furthermore, the human patches to be placed into the background images have to be extracted from the images. The generated images show some artefacts like sharp edges from the stitched elements and the stitched humans do not have shadows. Additionally, some humans that are automatically placed are located at unrealistic locations like on top of trees. This problem could be mitigated by specifying regions in which humans are allowed to be placed. This, however, would increase the manual effort needed to generate the images drastically. It remains unclear, however, how important these factors are and how large their influence is, if any.

**Figure 10:**
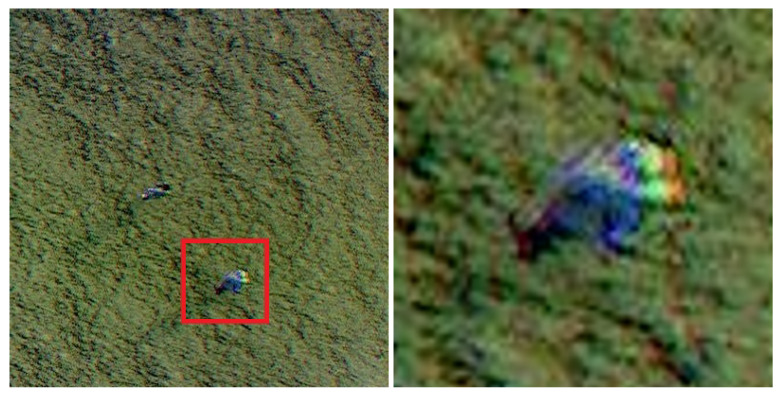
Example of blurred color gradients for small objects, when zoomed in. (**Left**): part of the original image, (**Right**): blurred color gradient after zooming into the marked area.

The generation of Class-3 synthetic images required the most effort of the three approaches. Although the Unreal Engine ecosystem provides a huge amount of simulation environments, 3D models of humans as well as tools to extract images, it requires some coding effort to put all these components together as described above. Furthermore, different rendering artefacts could be observed like blurred color gradients, especially for humans rendered as small objects of a scene. An example is given in Figure 10. Similar to the generation of Class-2 synthetic images, the automatic placement of humans locates some of them in unrealistic positions, e.g., on top of trees. Furthermore, people placed on inclined areas sometimes fall to the ground because of the gravitation model within the Unreal Engine resulting in people laying on the ground.

In general, it can be observed that the higher the class, the more effort is needed to generate synthetic data. In theory though, this effort is justifiable as higher classes allow more variation in the generated data which helps to increase completeness. However, the results show that this is not necessarily the case.

### 6.3. Usage of Synthetic Data to Increase Coverage of the ODD Parameter Altitude

From the results, it can be seen that the addition of synthetic images to improve the completeness of the dataset regarding altitude, when no real data are available, leads to better object detection performances in the considered use-case. The highest effect can be seen when adding Class-1 synthetic images. The trend here is that more data leads to better results. The results may even be improved further when more Class-1 data are added to the training set. In the experiments, an increase in AP@[IoU=0.5] from 0.27 to 0.44 was achieved in the ODD ranges in which no real-world images were available. These results were achieved without decreasing the performance in the other ODD ranges but even slightly improved them.

Small amounts of Class-2 and Class-3 data also give minor improvements. When more data of these classes are added, the results become worse or do not show any influence on the model performance on the altitude where no real data are available during training. On the images recorded at an altitude of 70 m or less, for which real data were available during training, the addition of the Class-2 and Class-3 data also does not show a significant influence but seems to lead to slightly worse results in some cases.

Overall, synthetic images seem suitable to increase the coverage of the ODD parameter altitude when not enough real-world images are available.

### 6.4. Domain Fitting and Overfitting

Although Class-1 synthetic images only allow us to introduce a limited amount of variation, they achieved the best results for the considered use-case.

As shown for the considered use-case, the addition of Class-1 synthetic images leads to the best results when the ODD cannot be covered fully with real-world images during training. A possible reason for this comes from the observation that the images in the used dataset all look very similar and are recorded at the same grassy park area. As a result, the Class-1 synthetic images show the people in similar positions as the test images recorded at the higher altitude. Therefore, the Class-1 synthetic images may lead the model to not only learn to detect humans but also their preferred positions in the area. This is not the case for the Class-2 and Class-3 synthetic images. They give the model incentives to only learn the human features leading to more robust detectors. This is also affirmed by the results on the Heridal dataset where Class-1 images led to the worst results. In general, this phenomenon may be considered as overfitting. But, it may also be considered inherent to the tasks of increasing ODD coverage with respect to the altitude, as these real images and the humans are considered the ODD, and therefore, the intended area of use. Therefore, one may say that the model is “well adjusted” to the situation and task, i.e., fits the data and domain nicely. The evaluation on the Herdial dataset also shows that, for other parameters, the higher synthetic data classes may be better and may help the model to generalize better when other ODD parameters are considered.

A potential reason that the model trained on Class-3 synthetic data performs worse than Class-2 synthetic data on the Heridal dataset may be that the simulated images are too different from real-world images. This phenomenon is also known as *sim-to-real gap* and sometimes found in the literature as described above. In a simulation environment, it is possible to generate very diverse images from the correct altitude but they may not represent the image domain well enough. Unwanted differences in appearances may prevent the models from learning features that generalize well to the real world. In contrast, Class-1 synthetic images may not be able to generate data with as many variations but represent the real image domain much better.

### 6.5. Limitations of the Approach

Using Class-1 synthetic images led to the highest improvement in model performance for the considered use-case. Our hypothesis is that this is the case because they are most similar to the real-world test data. However, this is only true for the considered use-case and may not be the case for other ODD parameters. This is especially true as Class-1 images have the strong limitation that they can only be generated if real-world data are available that can be transformed so that it represents the missing data. In the case of the ODD parameter altitude, this approach was well applicable, but this may not be the case for other ODD parameters. For example, Class-1 images cannot represent other geographical locations.

Class-2 data allows us to represent more ODD parameters and, e.g., could also be used to cover different geographical regions under the condition that real background images of these regions are available. Class-3 synthetic data allows us to introduce the most amount of variation and coverage of any possible ODD parameter in theory. However, as can be seen from the results, the generation approach for Class-3 data followed in this work has not succeeded in exploiting these advantages. This may be at least partially attributed to the domain gap, i.e., differences in appearance from the rendered images compared to the real-world ones. Nevertheless, Class-2 and Class-3 already show hints in Table 3 that they may be more suitable or at least can be suitable when applying the model to a different dataset that shows significant differences to the original one.

To conclude, it should be noted that there is a risk when using synthetic training data in safety-critical systems as it differs from the data the model will face when deployed. As explained above, current research suggest that real-world images lead to the best model results and should be used when available. However, synthetic images show promising results in this work and help to improve the model when not enough real-world data to cover the whole ODD are available. As a result, future research in this direction is strongly suggested. Whether the improvements and the overall performance of the model are good enough has to be evaluated during the certification of the whole aircraft system in which the ML component will be integrated.

## 7. Conclusions and Future Work

In this work, results from a case-study exploring the use of synthetic training data to increase the coverage of the ODD parameter *altitude* in the training dataset for human detection are presented. The generation of Class-1, Class-2 and Class-3 synthetic images is described and the generated data are used to increase the ODD coverage. The results indicate that the extension of the training dataset with additional synthetic data is promising in regards to the overall performance of an object detector. Within the addressed problem domain of detecting humans in low-altitude aerial images, as taken by onboard cameras of UAS, our results indicate that Class-1 synthetic data are best suited and lead to better overall performance. Particularly when using Class-1 synthetic images, a performance increase in AP@[IoU=0.5] from 0.27 to 0.44 was achieved in the real-world test dataset. These are very promising results with regard to reducing the amount of real-world data needed to cover the whole ODD during training. In contrast, the experimental results from using Class-2 and Class-3 synthetic data showed only marginal improvements or even a decrease in the performance. Hence, their potential benefits, especially the need for less real-world data, could not be leveraged. A plausible cause for the loss of performance is the domain shift introduced to the training dataset with the addition of Class-2 and Class-3 data. Furthermore, we discussed how these results could transfer to other ODD parameters like a geographic region.

The results show that synthetic data may be used to increase ODD coverage; however, care must be taken in regard to the quality and kind of synthetic data. For rather narrow ODDs, as considered in this work, Class-1 data may be best suited due to the low risk of introducing a significant domain shift that would lead to a loss of performance. However, this class of synthetic images cannot be used to increase the coverage of all ODD parameters as the real-world data may not be able to be transformed to represent the desired properties. For the use of Class-2 and Class-3 data, the wideness or narrowness of the ODD, in other words the degree of specialization required from the ML algorithm, may affect the amount of effort required to generate data that do not introduce unintentional domain shifts to the training dataset.

In future work, this interplay between the design domain and data requirements shall be further analyzed. Also, the impact of domain shifts and the sim-to-real gap, in general, are subjects to future research. In this context, domain adaptation techniques may be considered to reduce their effects. When looking at the considered use-case of human detection for emergency landing site detection, future work may include the detection of humans in different poses like laying and sitting, as well as increasing the overall system performance to bring it closer to reaching a performance level that may be deployed to real-world aircraft.

## Figures and Tables

**Table 1:** AP@[IoU=0.5] of the models on the real-world images captured at an altitude higher than 70 m. Results are averaged over 3 training runs. The bold number marks the best overall result.

	Fraction of Added Synthetic Data				
**Training Dataset**	**0.1**	**0.25**	**0.5**	**1.0**	**1.5**
Real (baseline)	--------------------------------- 0.27 ---------------------------------				
**Real + Class-1**	0.39	0.38	0.38	0.39	**0.44**
Real + Class-2	0.29	0.25	0.25	0.24	0.25
Real + Class-3	0.28	0.27	0.26	0.27	0.25

**Table 2:** AP@[IoU=0.5] of the models on the real-world test images captured at an altitude lower than 70 m. Results are averaged over 3 training runs. The bold number marks the best overall result.

	Fraction of Added Synthetic Data				
**Training Dataset**	**0.1**	**0.25**	**0.5**	**1.0**	**1.5**
Real (baseline)	--------------------------------- 0.56 ---------------------------------				
**Real + Class-1**	0.57	0.57	0.56	0.57	**0.58**
Real + Class-2	0.54	0.56	0.55	0.55	0.54
Real + Class-3	0.53	0.55	0.57	0.55	0.55

**Table 3:** Results on a subset of the Heridal dataset [29] with similar bounding box sizes as $X_{\mathrm{higher}}$. Results are averaged over 3 training runs. The bold number marks the best overall result.

Training Dataset	AP@[IoU = 0.5]
Real (baseline)	0.05
Class-1	0.15
**Class-2**	**0.30**
Class-3	0.26

## Data Availability

The PeopleOnGrass dataset is a third-party dataset and is publicly available at https://uni-tuebingen.de/fakultaeten/mathematisch-naturwissenschaftliche-fakultaet/fachbereiche/informatik/lehrstuehle/kognitive-systeme/projects/avalon/ (last accessed on 31 August 2023), see [16]. The Heridal dataset is a third-party dataset and is publicly available at http://ipsar.fesb.unist.hr/HERIDAL%20database.html (last accessed on 4 September 2023), see [29]. The Drones4Good dataset as well as the synthetic datasets are available on request from the corresponding author.

## Abbreviations

The following abbreviations are used in this manuscript:

AI	Artificial Intelligence
AP	Average Precision
CoDANN	Concepts of Design Assurance for Neural Networks
ConOps	Concepts of Operations
DLR	German Aerospace Center
EASA	European Union Aviation Safety Agency
IoU	Intersection over Union
ML	Machine Learning
OD	Operational Domain
ODD	Operational Design Domain
UAS	Unmanned Aircraft System
